# Newly discovered dimensional effects of electrodes on liquid crystal THz phase shifters enable novel switching between in-plane and out-of-plane

**DOI:** 10.1038/s41598-022-07832-x

**Published:** 2022-03-31

**Authors:** Masahito Oh-e, Deng-Yun Zheng

**Affiliations:** Institute of Photonics Technologies, Department of Electrical Engineering, National Tsing Hua University, 101 Sec. 2 Kuang-Fu Road, Hsinchu, 300044 Taiwan

**Keywords:** Optics and photonics, Optical materials and structures, Liquid crystals, Materials science, Soft materials, Liquid crystals, Optical physics, Terahertz optics

## Abstract

To unveil a novel switching mechanism in liquid crystal (LC)-based phase shifters for the THz range, we analyse how the dimensions of the electrode structures enable a new type of switching, namely, THz in-plane and THz out-of-plane (TIP–TOP) switching. Specifically, we determine how varying these electrode dimensions influences the LC in-plane states with the corresponding phase shifts by calculating these effects in virtual devices. Interestingly, we found that significant dimensional effects of the in-plane electrode structures statically and dynamically influence the phase shift and response time of LC switching. Analysing the electromagnetic fields in the TIP–TOP cell clearly reveals that these dimensional effects are due to changes in the electric field strengths caused by lateral bus-line electrodes that were originally assumed not to contribute to the switching. Further, we discover that the ultimate dimensional effect produces a novel type of LC switching, which results in hexadirectional switching between the initial, intrinsic in-plane, and out-of-plane reorientations of the LCs, suggesting a broader range of phase shifts while maintaining a rapid response.

## Introduction

Advancing terahertz (THz) modulation technology is a prerequisite for high-capacity, high-speed broadband wireless communication, security surveillance, high-resolution medical imaging, and materials characterization^1–4^. To this end, many quasi-optical components must be developed for THz devices, such as phase shifters^5–14^, filters^15–17^, phase gratings^18,19^, and polarizers^20–22^. For example, arrays of phase shifters can be used to form THz beams. In such device components, tunability is an important characteristic; in fact, tunable phase shifters have been demonstrated based on optically or electrically controllable carrier concentrations in quantum-well structures^23–25^. These quantum-well-based THz phase shifters, however, have yet to provide sufficient phase shifts and must be operated at temperatures far below room temperature. Meanwhile, metamaterial structures provide rapid-switching phase shifters, but they normally suffer high insertion losses^26–28^. By contrast, liquid crystals (LCs) play a promising role in achieving devices that can be tuned using external fields at room temperature while maintaining low insertion losses; for example, some phase shifters using LCs have been successfully demonstrated in the THz frequency range^12–14,29^.

The cell gap required for THz phase shifters depends on the birefringence of the LCs and the quantity of phase shifts. The complex refractive indices of LCs, such as 4′-n-pentyl-4-cyanobiphenyl (5CB) at room temperature, were determined by THz time-domain spectroscopy^30,31^. 5CB exhibits a significantly large birefringence with small extinction coefficients at frequencies of approximately 1 THz. Magnetic and electric fields enable controlling the birefringence in a sandwiched LC cell, which realizes phase shifts larger than 2π at 1 THz^5,6,14,29^. In principle, however, a large cell gap in the range of hundreds of micrometres is required to attain enough retardation and thus sufficient phase modulation at THz frequencies. Therefore, an extremely slow response is inevitable for this type of unusually thick LC device, in contrast with thinner ones, in this optical frequency region. Although one type of switching under an external field is relatively fast on timescales of seconds or fractions of a second, the other under no field requires tens or hundreds of seconds or more, which is a serious drawback for LC-based THz devices. Polymer network LC technology has been implemented to help reorient the LCs, but it did not adequately improve the switch-off time^32^.

Meanwhile, as LC technology continued to develop, a new electrode design for bidirectionally switching LCs was proposed, wherein LCs are actively driven using electric fields, including the switching-off process at THz frequencies^33,34^. In this bidirectional switching, the electrodes are composed of finger-type electrodes that produce in-plane (i.e., parallel to the substrate) electric fields, which are combined with other multiple grating-type electrodes that produce out-of-plane (i.e., vertical to the substrate) electric fields. The same electrode pattern is created on each glass substrate that constitutes an LC cell, and each pattern exactly mirrors that on the opposite substrate. The authors named this type of switching with this electrode design ‘THz in-plane and THz out-of-plane (TIP–TOP)’ switching^33,34^. This TIP–TOP switching enables switching the LCs in both directions, thereby allowing the flexible control of the on- and off-times by applying a voltage. It therefore attains a response time 100 times faster than that of conventional devices that rely on the passive relaxation of the LCs under a potential difference of 100 V (peak-to-peak voltage). The performance of a TIP–TOP-switching phase shifter is successfully demonstrated, and a controllable phase shift of 35° at 2 THz is realized using a direct current with a bias of 100 V to switch between the in-plane and out-of-plane states. The active switching speed is between 10 and 470 ms, which is 100 times faster than conventional relaxation times without applied fields. Although TIP–TOP switching improves some device characteristics, its detailed mechanisms such as how the electric fields are distributed and how the LCs respond to the fields are not necessarily understood.

In this study, we clarify some of these mechanisms, and we report an intriguing new feature, which we discovered while analysing the effects of the TIP–TOP electrode structures based on our interest in the further development of this type of switching for THz phase shifters. We were originally interested in why the driving voltage remains high and how to decrease it. Within certain cell parameters, the threshold voltage for LCs to be driven correlates with the cell gap and electrode distance; however, we observed that another parameter unexpectedly affected the switching behaviour which would be worth considering to further advance the quest for a novel type of switching in LC-based phase shifters in the THz range.

## Methods

All electro-optical characteristics of the TIP–TOP-switching LC-based phase shifter in this study were calculated using modelling and evaluation software for LCD designers, LCDMaster 3D^35^, which includes a computer-aided design module for LC cells and their electrode structures, LC molecular profiles for various applied voltages at given boundary conditions using the finite-element method^36^ in three dimensions, and optical characteristics deduced from the LC orientations. A complementary electromagnetic field analysis was performed using COMSOL Multiphysics^37^, which enabled deducing the electromagnetic fields in space using the finite-element method on the same model with the geometries defined for the TIP–TOP structures in three dimensions.

**Figure 1:**
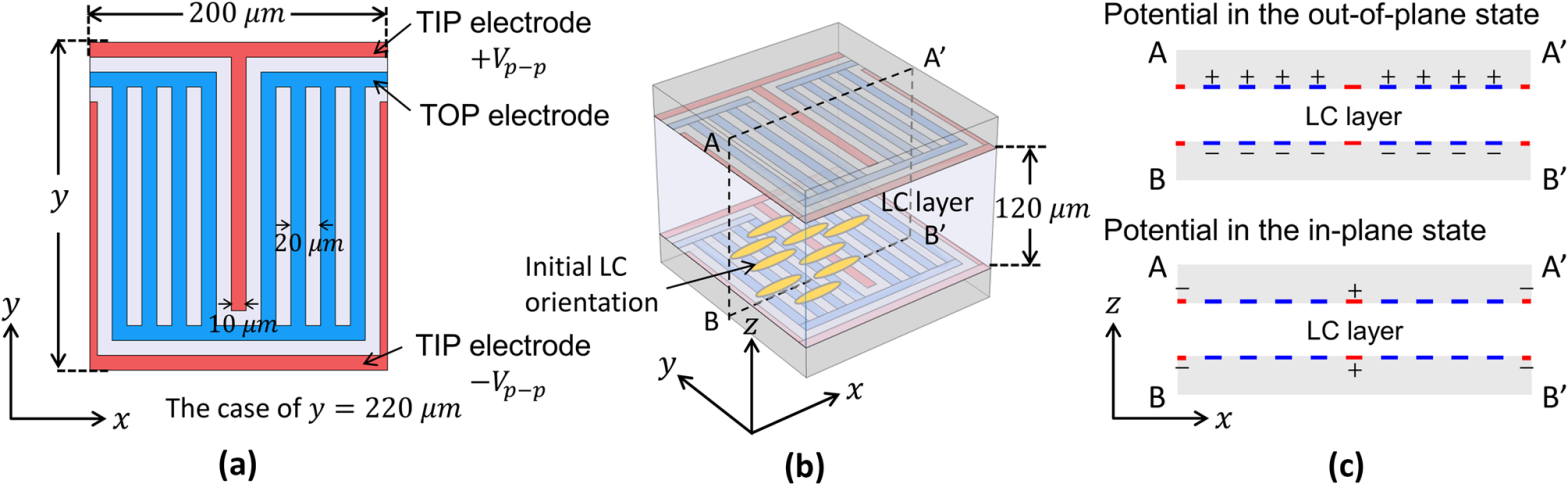
Schematics showing the geometry of the unit cell used in our calculations. (**a**) Electrode structures on each substrate. (**b**) Unit cell structure. (**c**) Potential applied to the electrodes along cross-section AA′BB′ illustrated in (**b**).

We first followed the original electrode layout of the TIP–TOP switching demonstrated by Ung et al.^34^. Figure 1a,b show the geometry of a cell structure, wherein the TIP and TOP electrodes are finger- and grating-type structures, respectively, superimposed on the inner surfaces of the top and bottom substrates and separated by a gap filled with LCs. The finger- and grating-type electrodes extend in the *y-*direction, while the entire structure periodically repeats in the *x-*direction. The finger-type TIP electrodes on each substrate apply in-plane fields, while the grating-type TOP electrodes apply out-of-plane fields. Figure 1c shows the potential applied to the electrodes for the out-of-plane and in-plane states. In principle, the in-plane and out-of-plane fields reorient the LCs with positive dielectric anisotropy parallel and perpendicular to the substrates, respectively; however, the in-plane fields do not switch the LCs in-plane because they are initially oriented perpendicular to the TIP electrodes. Rather, the TIP electrodes switch the LCs out-of-plane in the immediate vicinity of the electrodes. The TOP electrodes are formed with a width/interval of 10/20 μm, and positive and negative voltages are applied separately to the pair of TOP electrodes on the two substrates for the TOP switching. A pair of TIP electrodes are laid out on each substrate with a width/interval of 10/100 μm, and they are alternatingly connected to a bias to provide positive and negative potentials for the TIP switching. When the TIP switching is on, the TOP electrodes remain floating.

To model the LC cell, the cell gap for this TIP–TOP switching was set to 120 μm and assumed to be filled by an LC material (RDP-94990, Dainippon Ink & Chemicals Corporation, Tokyo, Japan). RDP-94990 has refractive indices of $n_{\parallel } = 1.77, n_{ \bot } = 1.57$ at 550 nm and has demonstrated a relatively high birefringence of ${\Delta }n_{THz} = 0.2$ at THz frequencies^38^. The splay, twist, and bend elastic constants were assumed to be $K_{1} = 4.73$, $K_{2} = 1.60$, and $K_{3} = 3.60{\text{ pN}}$, respectively. The dielectric anisotropy and rotational viscosity were $\Delta \varepsilon = 6.5$ and $\gamma_{1} = 0.139 {\text{ Pa}} \cdot {\text{s}}$, respectively. The LCs were initially aligned along the *x-*direction, which is parallel to the substrate plane and perpendicular to the direction in which the electrodes extended, as shown in Fig. 1. The cell was assumed to be sandwiched by a pair of crossed polarizers, one polarization axis of which was $45^\circ$ to the *x-*axis to attain the maximum modulation.

## Results and discussion

In their previous study, Ung et al.^34^ measured and calculated the phase changes of the TIP–TOP cells at 2 THz, and the phase shifted by almost $57^\circ$ when they switched the bias of the TOP electrode from the ground (GND: no voltage and hence the initial state) to the out-of-plane state of 100 V (peak-to-peak voltage); further, it shifted by $\sim 29^\circ$ when switching from GND to the in-plane state of 100 V. In addition, they demonstrated a 29°–57° phase shift when switching from the in-plane state of 100 V to the out-of-plane state of 100 V. Further, they achieved bidirectional switching, i.e., in-to-out and out-to-in switching, both of which were active transitions under the electric fields rather than passive relaxation after removing the electric fields, thereby reducing the response time by a factor of 100.

Using the same layout of electrodes and TIP–TOP cell structures as those in the previous study^34^, our calculations can replicate these phase shifts. The software package LCDMaster 3D provides a platform wherein the LC directors and electric potentials can be numerically solved in finely discretized space and time using the finite-element method^36^. The equation of motion for the LC director ${\varvec{n}}$ is expressed as^38,39^:

1 $${\upgamma }_{1} \frac{{\partial n_{i} }}{\partial t} = \left( {\frac{\partial F}{{\partial n_{i,j} }}} \right)_{,j} - \frac{\partial F}{{\partial n_{i} }} + G_{i} + \lambda n_{i} \ , \quad i, j = x, y, z \ ,$$

where Einstein notation is used, and the comma immediately before the subscript indicates the partial differential of the coordinates with respect to the given coordinate of the subscript. Equation (1) represents the balance between three types of torques that are generated by the electric field, viscous resistance, and elastic restoring force. ${\uplambda }$ is the undetermined multiplier under the condition $n_{j} n_{j} = 1$. $F$ is the increase in free energy due to the elastic deformation expressed by $K_{1}$, $K_{2}$, and $K_{3}$ of the LC directors. $G$ is the torque generated by the electric field, expressed as $G_{i} = \Delta \varepsilon n_{j} E_{j} E_{i} = \Delta \varepsilon n_{j} \phi_{, j} \phi_{, i}$, where $\phi$ is the potential. From Poisson’s equation, $\left( {\varepsilon_{ij} \phi_{,j} } \right)_{,i} = 0$ can be derived using the dielectric tensor $\varepsilon_{ij} = \varepsilon_{ \bot } \delta_{ij} + \left( {\varepsilon_{\parallel } - \varepsilon_{ \bot } } \right)n_{i} n_{j}$. These equations enable numerically deducing ${\varvec{n}}$ and $\phi$ under the periodic boundary condition for the *x-*direction and Neumann’s boundary condition for the *y*- and *z*-directions. Further, the characteristics of the polarized light propagation in the obtained LC director distribution can be simulated using the 2 × 2 matrix method^40,41^.

**Figure 2:**
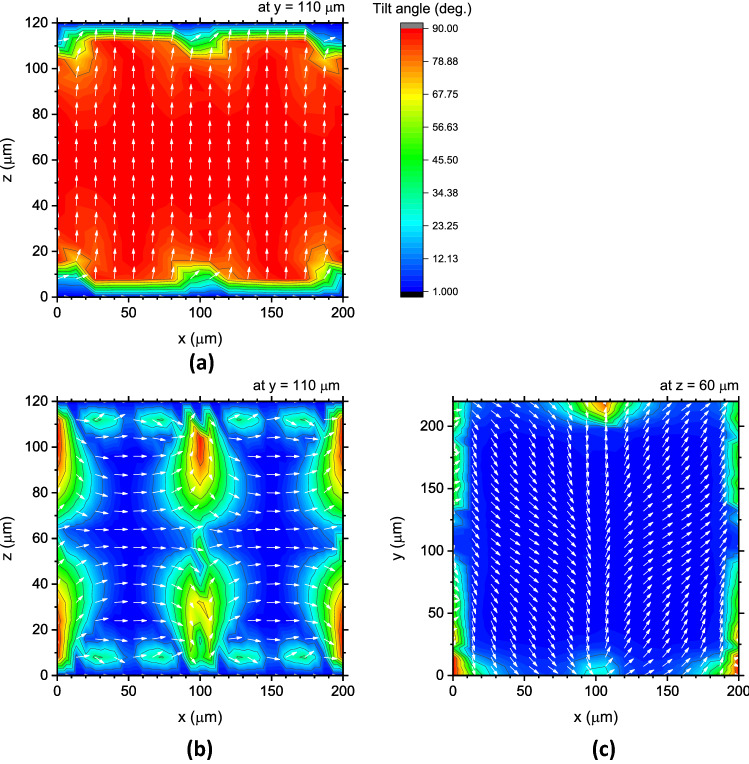
LC director distributions in the unit cell of x: 200 × y: 220 µm; (**a**) in the x–z cross-section at the centre of the y-axis under the out-of-plane state of 100 V, and (**b**) in the x–z cross-section at the centre of the y-axis and (**c**) in the x–y horizontal section under the in-plane states of 100 V. The colours indicate the tilt angle from the surface, and the white arrows are LC directors.

Figure 2 shows the LC director distributions in the TIP–TOP cell that we calculated under the out-of-plane and in-plane states of 100 V. These calculations were converged for the case of the unit cell with dimensions of 200 × 220 μm under an error criterion of 10^−5^ using a tetrahedral mesh with six sides of 2.0 μm in length. The obtained LC director distribution in the rectangular cross-section at the centre of the *y*-axis, i.e., at *y* = 110 μm are indeed replicated in the same manner as in the previous study. Using the LC director distributions, the phase shifts when the state changes from GND to the out-of-plane state of 100 V and from GND to the in-plane state of 100 V were deduced to be $53.83^\circ$ and $34.07^\circ$ at 2 THz, respectively, which are almost equivalent to those in the previous study^34^. Starting from this well-reproduced result, we performed further calculations on the THz TIP–TOP phase shifter under the same calculation conditions.

**Figure 3:**
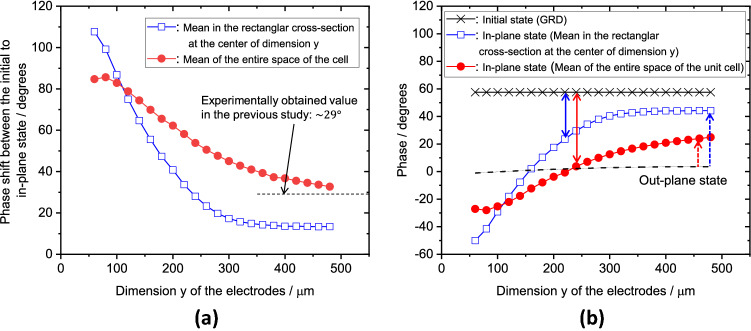
(**a**) Phase shifts at 2 THz from GND to the in-plane state of 100 V and (**b**) phase values of each state as functions of the electrode length *y*.

Surprisingly, varying the dimensions of the electrodes along the *y-*axis revealed significant changes in the phase shift from GND to the in-plane state of 100 V. Figure 3 shows these phase shifts at 2 THz and the phase values of each state as a function of the length of the electrode along the *y*-axis. Two series of data are plotted in Fig. 3a: one is the average phase shift in the rectangular plane of the cross-section at the centre of the *y*-axis, and the other is the counterpart for the entire space of the unit cell. Both plots indicate that the phase shift from GND to the in-plane state of 100 V decreases as the electrode length *y* increases. Conversely, the shorter the length in the *y*-dimension, the larger is this phase shift.

Initially, this was an unexpected result, as the in-plane fields considered in the original TIP–TOP structure were determined by a pair of alternating finger-type electrodes, and the switching of the LC is approximated by $E_{c} \times \ell \propto \sim \pi \ell /d$ ^42^, where $E_{c}$ is the threshold electric field strength, $\ell$ is the finger-type electrode distance, and $d$ is the cell gap. Although the in-plane fields are created by the pair of alternating finger-type electrodes with positive and negative potentials along the *x*-axis, the LCs do not switch in-plane because of the initial LC orientation; rather, they reorient out-of-plane near the electrodes, as shown in Fig. 2. However, as the length *y* decreases, the LCs increasingly experience another in-plane field along the *y-*axis, which gradually strengthens because the bus lines of the TIP electrodes lie along the *x-*axis. When this in-plane field along the y-axis increases, it causes intrinsic in-plane switching. In addition, as the total electric field increases, the LCs become more strongly deformed, thereby producing a larger phase shift.

In the two phase shift profiles in Fig. 3a, the mean phase shift along the rectangular cross-section at the centre of the *y*-axis and that of the entire space of the cell both decrease as the dimension *y* increases. However, these mean phase shifts decrease to different degrees with increasing *y*. Moreover, they are not equal at any value of *y*. This finding suggests that the phase shifts in the space of the unit cell are not uniform, and thus, the switching of the LC caused by the in-plane fields is spatially inhomogeneous within the cell. By contrast, the transition from GND to the out-of-plane state (the electric field is perpendicular to the substrates) is uniform; therefore, the phase shifts in the entire cell space and the rectangular cross-section at the centre of the *y*-axis are approximately equivalent to the corresponding mean phase shifts, which are 55.82° for the entire cell and 53.83° along the cross-section.

The previous study^34^ experimentally demonstrated a phase shift from GND to the in-plane state of ~ 29°, which presumably corresponds to extrapolating the decreasing trend in the phase shift for the mean of the entire space toward the larger length in the *y-*dimension. Indeed, as the length *y* increases, extrapolating the plots of the mean of the entire space approaches the experimentally obtained value, as shown in Fig. 3a. According to Ung et al.^34^, after switching from GND, the in-plane state was demonstrated to be an intermediate state; however, this state could be changed or tuned by varying the electrode length *y*. Further, the results suggest that shortening the length *y* can also increase the phase shift from GND to the in-plane state to be larger than the out-of-plane phase shift from GND, i.e., $\sim 57^\circ$. The solid double-headed arrows in Fig. 3b indicate the tunable range of the phase shifts in the in-plane state. The shorter length *y* indeed enables broadening the range of phase shifts between GND (i.e., the initial state) to the in-plane state, which can further exceed the phase shift of the out-of-plane state. The changes in the phase shifts because of the length of the electrode along the *y-*axis can be regarded as a dimensional effect.

To observe how this dimensional effect is actually caused by changes in electric field strengths and their distributions, we analysed the potential and electric field distributions in the unit cell while varying the length *y*. The required quantities were obtained together with the LC director distributions. Complementary calculations were performed using COMSOL Multiphysics with the same model as the one illustrated in Fig. 1, an error criterion of 10^−5^, and a six-sided tetrahedral mesh with a length of 0.1–2.0 μm, under which reasonably moderate changes in the field strength along the *z*-axis were attained. Otherwise, the weak electric field along the *z-*axis did not exhibit a smooth trend.

**Figure 4:**
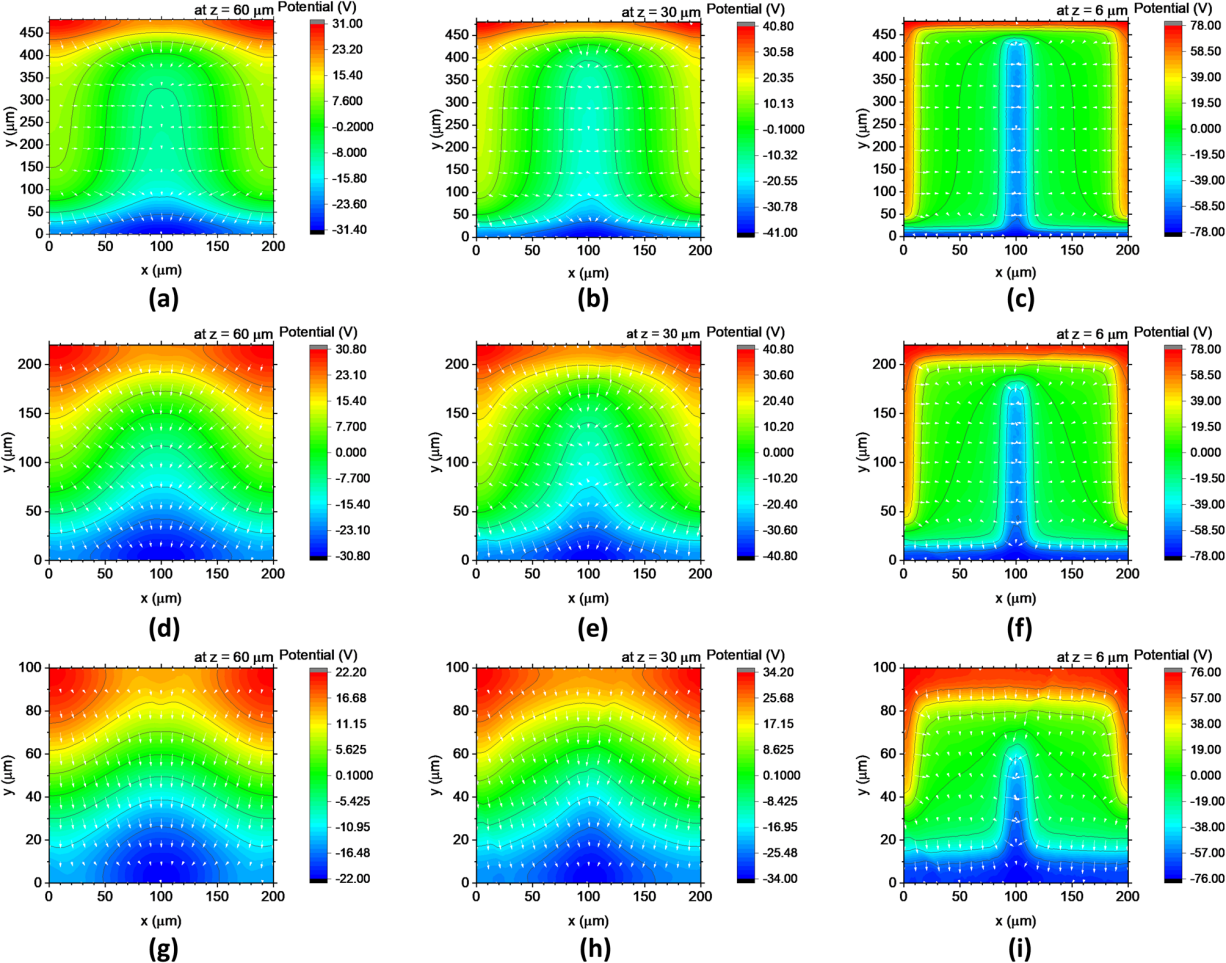
Potential and electric field distributions in the horizontal sections of the unit cells with dimension of x = 200 µm and y = (**a**–**c**) 480 µm, (**d**–**f**) 200 µm, and (**g**–**i**) 100 µm in the in-plane state of 100 V. (**a**), (**d**), and (**g**) are at *z* = 60 μm, (**b**), (**e**), and (**h**) are at *z* = 30 μm, and (**c**), (**f**), and (**i**) are at *z* = 6 μm. The white arrows in each graph indicate relative electric fields.

This analysis allowed us to correlate the tendency of the average phase shift, and thus the LC orientation, with that of the electric field distributions in the space of the unit cell. Figure 4 compares the changes in the potential and electric field distributions upon switching to the in-plane state of 100 V in some *x*–*y* horizontal sections for various lengths along the *y*-axis. The electric fields are perpendicular to the contour lines of the potential. The electric field component $E_{x}$ is dominant when *y* = 480 μm, except at both edges of the cell, where the bus lines of the TIP electrodes are located. As the length *y* decreases from *y* = 480 µm to $y = 220$ and *y* = 100 μm, the component $E_{y}$ more significantly affects the entire space in the *x*–*y* horizontal planes of the cell. Further, this effect is even stronger at *z* = 60 μm, which is the centre of the cell thickness, than that near the surface of the cell. $E_{x}$ hardly changes with *y*, while $E_{y}$ significantly increases, even at the centre in the *x*–*y* horizontal planes of the cells with shorter *y* dimensions. Therefore, shortening the cell along the *y*-axis drastically increases $\left| {E_{{{\text{total}}}} } \right|$ because of $\left| {E_{y} } \right|$, which thus becomes a driving force for switching the LCs in-plane. This switching starts from the centre of the cell thickness rather than near the surface.

**Figure 5:**
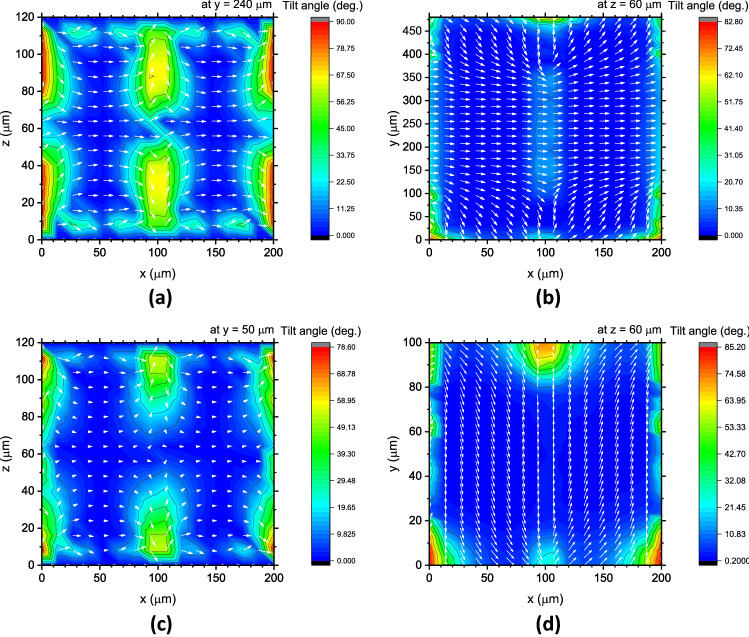
LC director distributions in the unit cells of $x$: 200 × $y$: 480 µm (top) and $x$: 200 × $y$: 100 µm (bottom); (**a**), (**c**) in the *x*–*z* cross-section at the centre of the $y$-axis, and (**b**), (**d**) in the *x*–*y* horizontal-section under the in-plane states of 100 V. The colours indicate the tilt angle from the surface, and the white lines are LC directors.

In fact, the intrinsic in-plane switching of LCs occurs when $\left| {E_{y} } \right|$ becomes comparable to $\left| {E_{x} } \right|$ as the length *y* decreases. Figure 5 compares the distributions of the LC directors in the in-plane state of 100 V for $y = 480$ and $100$ μm. In the latter case, the LC directors in almost all areas of the horizontal *x*–*y* plane are clearly oriented along the *y-*axis with no disclination, while the counterparts in the former case reorient along the *x-*axis, except for the area close to the electrodes lying along the *x-*axis. Figure 2 shows the case of *y* = 220 μm, which lies between the two cases in Fig. 5. These results suggest that as the length *y* decreases, the intrinsic in-plane switching indeed occurs, which orients LCs along the *y-*axis. This in-plane-field-induced orientation is perpendicular to the initial state, and therefore, the phase becomes opposite to that of the initial state. The negative phases shown in Fig. 3b correspond to such LC reorientations induced by the in-plane fields along the *y-*axis.

The dynamic responses of LCs are also significantly influenced by the dimensional effects of the in-plane electrode structures. The response time of the LC reorientation $\tau_{{{\text{off}}}}$ upon removing the electric field is proportional to the square of the cell gap, i.e., $\tau_{{{\text{off}}}} \propto d^{2} /K$ ^43^, where $d$ is the cell gap, and $K$ is the elastic constant of the LC. For THz phase shifters, as long as the cell gap is several tens to hundreds of micrometres, the response time is extremely slow, unlike in LC displays, wherein the cell gap is typically only a few micrometres. In this sense, the bidirectional switching using electric fields demonstrated by Ung et al.^34^ is the correct pathway to realize an LC-based THz phase shifter, because the response time to an electric field $\tau_{{{\text{on}}}}$ is inversely proportional to the square of the electric field $E$, i.e., $\tau_{{{\text{on}}}} \propto 1/E^{2}$ ^43^.

**Figure 6:**
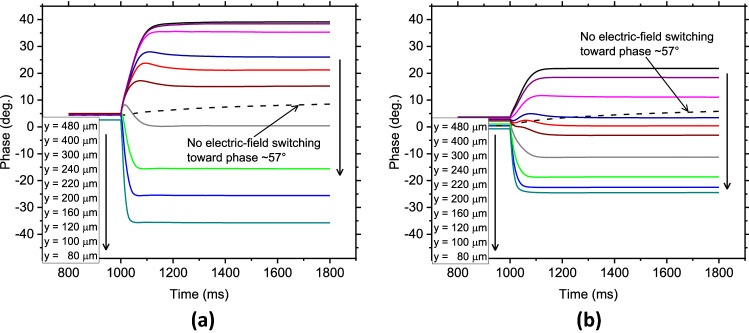
Dynamic phase changes calculated at 2 THz when each cell is switched from the out-of-plane state of 100 V to the in-plane state of 100 V for various values of *y*; (**a**) Mean phase changes in the *x*–*z* cross-section at the centre of the *y*-axis. (**b**) Mean phase changes of the entire unit cell space. For the first 1000 ms, the out-plane state of 100 V was applied to each cell, and the potential was switched to the in-plane state of 100 V at 1000 ms. The dotted line shows the case without electric field switching in the cell with y = 100 μm, meaning switching only from the out-plane state of 100 V to GND.

Figure 6 compares the dynamic changes in the phase at 2 THz from the out-of-plane state of 100 V to the in-plane state of 100 V for various cell lengths *y*. Specifically, a voltage of 100 V was applied between the TOP electrodes for 1 s, followed by applying a voltage of 100 V between the TIP electrodes, which is referred to as out-to-in switching and corresponds to the transitions indicated by the single-headed arrows in Fig. 3b. The equilibrated orientations of the LC directors for every 20 µs were calculated, from which the phase through the LC cell was deduced. The other calculation conditions were the same as those for the static cases. In these dynamical calculations, LC directors were basically perpendicular to the substrate as the starting out-of-plane state, except for the space between the TOP electrodes; thus, the phase of the starting state was almost zero. As a reference, a case wherein the electric field was not switched was also calculated, in which the initial potential at the out-of-plane state was simply removed (i.e., GND), and the LC directors relaxed back to their initial alignment corresponding to a phase of $\sim 57^\circ$.

Overall, out-to-in switching transitions between the statically analysed states were observed, albeit with some phase shifts. The results suggest that all transitions can be driven by appropriately changing the potential of the electrodes. More importantly, the transitions occur under the influence of electric fields, thereby producing more practical response times than those that occur after simply removing the electric fields; in fact, the calculated case without electric field switching, which corresponds to the transition in conventional LC-based THz phase shifters, was ~ 100 times slower. Further, the dimensional effects still appeared, namely, the shorter the length in the *y-*dimension, the faster the response time. Defining the response time as the time required to change the phase by 90% gives times of 95, 65, and 40 ms for *y* = 480, 220, and 100 μm, respectively. Therefore, the dimensional effect can also dynamically influence the LC responses, which is reasonable when considering that the electric-field strength significantly governs the response time.

For various values of *y*, the transitional response profiles for the cases of large and small *y* change to smoothly follow the step function of the potential, whereas those for intermediate *y* overshoot the target phase while exhibiting a weakly overdamped response. This behaviour is probably not due to the backflow effect^44,45^, because switching is controlled under a strong electric field, and further, the observed overdamped response is limited to only the intermediate *y* cases. Although this overshooting-like response should be analysed in detail, it is presumably a result of the competition between $\left| {E_{x} } \right|$ and $\left| {E_{y} } \right|$, which are inhomogeneously distributed in the space of the unit cell.

The dimensional effect becomes pronounced when the length *y* is comparable to or less than the distance between the electrodes that provide an in-plane field along the *x-*axis. This effect is due to the lateral bus lines of the TIP electrodes, the potential of which would be originally considered noise or would be assumed not to contribute to switching. Interestingly, discovering this dimensional effect allows us to utilize the electric field along the *y*-axis, which effectively enables continuously switching the LC orientation between the three states: initial, out-of-plane, and intrinsic in-plane states. The initial state is the LC orientation without electric fields, the out-of-plane state arises when an electric field is applied vertically to the substrates via the TOP electrodes, and the intrinsic in-plane state occurs when a potential is provided to the TIP electrodes. In the last process, when $\left| {E_{y} } \right| > \left| {E_{x} } \right|$, the LC orientation changes from the out-of-plane state toward the intrinsic in-plane state; otherwise, the LC orientation returns to the initial state. In principle, the former therefore broadens the range of phase shift with respect to the initial state because of the change in the LC orientation.

Pursuing this dimensional effect allows us to separate the TIP electrodes into two parts for applying different in-plane fields along the *x*- and *y-*axes. Independently applying in-plane fields enables continuously switching the LC orientation between the aforementioned initial and intrinsic in-plane states. Therefore, mutual switching between the three states effectively becomes possible, although the dimensional effect indicates that the switching between the initial and intrinsic in-plane states using one set of TIP electrodes is achieved with passing through the out-of-plane state. In other words, this dimensional effect allows us to generate a new LC switching mode, which effectively allows hexadirectional switching between the initial, intrinsic in-plane, and out-of-plane states. In principle, utilizing the three states broadens the changes in phase shifts, and switching by applying electric fields maintains a faster response time. The range of phase shifts afforded by the novel LC switching mode for THz devices such as phase shifters can potentially be further improved by optimizing some device and material parameters, including even larger retardation using LCs with greater birefringence.

## Conclusion

We discovered the dimensional effects of TIP–TOP switching and found that these effects become remarkable when the electric field from the lateral bus-line electrodes has a large effect. These dimensional effects enable continuously switching LC orientation between the initial, intrinsic in-plane, and out-of-plane states, thereby providing a wider range of phase shifts while maintaining a raid response. By systematically analysing these effects, we were able to identify their mechanism, paving the way to the possibility of novel LC switching by exploiting these dimensional effects and manipulating the hexadirectional continuous switching between the three LC orientation states. Nevertheless, further efforts should be devoted to revealing the possibility of this novel switching mechanism to advance the quest for LC-based THz devices.

## Data Availability

Data underlying the results presented in this paper are not publicly available at this time but may be obtained from the authors upon reasonable request.

## References

[1] Ferguson B, Zhang X-C (2002). Materials for terahertz science and technology. Nat. Mater..

[2] Tonouchi M (2007). Cutting-edge terahertz technology. Nat. Photon..

[3] Yang Y, Mandehgar M, Grischkowsky D (2015). THz-TDS characterization of the digital communication channels of the atmosphere and the enabled applications. J. Infrared Milli. Terahertz Waves.

[4] Nagatsuma T, Ducournau G, Renaud CC (2016). Advances in terahertz communications accelerated by photonics. Nat. Photon..

[5] Chen C-Y, Hsieh C-F, Lin Y-F, Pan R-P, Pan C-L (2004). Magnetically tunable room-temperature 2π liquid crystal terahertz phase shifter. Opt. Express.

[6] Wu H-Y, Hsieh C-F, Tang T-T, Pan R-P, Pan C-L (2006). Electrically tunable room-temperature 2π liquid crystal terahertz phase shifter. IEEE Photon. Technol. Lett..

[7] Lin X-W (2011). Self-polarizing terahertz liquid crystal phase shifter. AIP Adv..

[8] Wu Y (2013). Graphene/liquid crystal based terahertz phase shifters. Opt. Express.

[9] Yang C-S, Tang T-T, Pan R-P, Yu P, Pan C-L (2014). Liquid crystal terahertz phase shifters with functional indium-tin-oxide nanostructures for biasing and alignment. Appl. Phys. Lett..

[10] Yang C-S (2015). Liquid-crystal terahertz quarter-wave plate using chemical-vapor-deposited graphene electrodes. IEEE Photon. J..

[11] Sasaki T, Noda K, Kawatsuki N, Ono H (2015). Universal polarization terahertz phase controllers using randomly aligned liquid crystal cells with graphene electrodes. Opt. Lett..

[12] Wang L (2015). Broadband tunable liquid crystal terahertz waveplates driven with porous graphene electrodes. Light Sci. Appl..

[13] Du Y, Tian H, Cui X, Wang H, Zhou Z-X (2016). Electrically tunable liquid crystal terahertz phase shifter driven by transparent polymer electrodes. J. Mater. Chem. C.

[14] Yang C-S (2019). High-transmittance 2π electrically tunable terahertz phase shifter with CMOS-compatible driving voltage enabled by liquid crystals. Appl. Sci..

[15] Chen C-Y, Pan C-L, Hsieh C-F, Lin Y-F, Pan R-P (2006). Liquid-crystal-based terahertz tunable Lyot filter. Appl. Phys. Lett..

[16] Gavdush AA (2020). Proof of concept for continuously-tunable terahertz bandpass filter based on a gradient metal-hole array. Opt. Express.

[17] Huang Y (2020). Actively tunable THz filter based on an electromagnetically induced transparency analog hybridized with a MEMS metamaterial. Sci. Rep..

[18] Lin C-J, Li Y-T, Hsieh C-F, Pan R-P, Pan C-L (2008). Manipulating terahertz wave by a magnetically tunable liquid crystal phase grating. Opt. Express.

[19] Pan C-L, Lin C-J, Yang C-S, Wu W-T, Pan R-P, Choudhury PK (2018). Liquid-crystal-based phase gratings and beam steerers for terahertz waves. Liquid Crystals: Recent Advancements in Fundamental and Device Technologies.

[20] Hsieh C-F, Lai Y-C, Pan R-P, Pan C-L (2008). Polarizing terahertz waves with nematic liquid crystals. Opt. Lett..

[21] Vasić B, Zografopoulos DC, Isić G, Beccherelli R, Gajić R (2017). Electrically tunable terahertz polarization converter based on overcoupled metal-isolator-metal metamaterials infiltrated with liquid crystals. Nanotechnology.

[22] Sasaki T (2017). Twisted nematic liquid crystal cells with rubbed poly(3,4-ethylenedioxythiophene)/poly(styrenesulfonate) films for active polarization control of terahertz waves. J. Appl. Phys..

[23] Libon IH (2000). An optically controllable terahertz filter. Appl. Phys. Lett..

[24] Kersting R, Strasser G, Unterrainer K (2000). Terahertz phase modulator. Electron. Lett..

[25] Kleine-Ostmann T, Koch M, Dawson P (2002). Modulation of THz radiation by semiconductor nanostructures. Microw. Opt. Technol. Lett..

[26] Chen H-T (2008). Hybrid metamaterials enable fast electrical modulation of freely propagating terahertz waves. Appl. Phys. Lett..

[27] Miao Z (2015). Widely tunable terahertz phase modulation with gate-controlled graphene metasurfaces. Phys. Rev. X.

[28] Dong DS (2015). Terahertz broadband low-reflection metasurface by controlling phase distributions. Adv. Opt. Mater..

[29] Li S (2020). Super terahertz phase shifter achieving high transmission and large modulation depth. Opt. Lett..

[30] Tsai T-R, Chen C-Y, Pan C-L, Pan R-P, Zhang X-C (2003). Terahertz time-domain spectroscopy studies of the optical constants of the nematic liquid crystal 5CB. Appl. Opt..

[31] Oh-e M, Yokoyama H, Koeberg M, Hendry E, Bonn M (2006). High-frequency dielectric relaxation of liquid crystals: THz time-domain spectroscopy of liquid crystal colloids. Opt. Express.

[32] Altmann K (2013). Polymer stabilized liquid crystal phase shifter for terahertz waves. Opt. Express.

[33] Ung, B. S.-Y. *et al.* Terahertz in plane and terahertz out of plane (TIP–TOP) switching of a liquid crystal spatial light modulator in *2014 39th International Conference on* Infrared*, **Millimeter,* and Terahertz waves *(IRMMW-THz)* 1–2. 10.1109/IRMMW-THz.2014.6956348 (IEEE Computer Soc., 2014).

[34] Ung BS-Y (2018). Towards a rapid terahertz liquid crystal phase shifter: Terahertz in-plane and terahertz out-plane (TIP–TOP) switching. IEEE Trans. Terahertz Sci. Technol..

[35] LCDMaster v. 10.3.0. www.shintechoptics.com. (Shintech, Inc., Yamaguchi, Japan).

[36] Reddy JN (2006). Introduction to the Finite Element Method.

[37] COMSOL Multiphysics v. 5.6. www.comsol.com. (COMSOL AB, Stockholm, Sweden).

[38] Park H (2012). Evaluating liquid crystal properties for use in terahertz devices. Opt. Express.

[39] Kitamura M (2002). Simulation technology for LCDs. EKISHO.

[40] Khoo I-C (2007). Liquid Crystals.

[41] Clark Jones R (1942). A new calculus for the treatment of optical systems IV. J. Opt. Soc. Am..

[42] Oh-e M, Kondo K (1995). Electro-optical characteristics and switching behavior of the in-plane switching mode. Appl. Phys. Lett..

[43] Oh-e M, Kondo K (1996). Response mechanism of nematic liquid crystals using the in-plane switching mode. Appl. Phys. Lett..

[44] Jewell SA, Sambles JR (2003). Backflow in the relaxation of a hybrid aligned nematic cell. Appl. Phys. Lett..

[45] Zhang Y-D (2019). Backflow effect enabling fast response and low driving voltage of electrophoretic E-ink dispersion by liquid crystal additives. Sci. Rep..

